# Correlations between atazanavir C_trough _and hyperbilirubinemia: a case report

**DOI:** 10.1186/1752-1947-3-9307

**Published:** 2009-12-01

**Authors:** Alessia Uglietti, Stefano Novati, Roberto Gulminetti, Renato Maserati

**Affiliations:** 1 HIV/AIDS Outpatient Clinic Infectious Disease Dept, Foundation "IRCCS Policlinico San Matteo Hospital", 27100 Pavia, Italy

## Abstract

**Introduction:**

Hyperbilirubinemia is a common side effect of the antiretroviral agent atazanavir but is generally reversible upon discontinuation of treatment. We used therapeutic drug monitoring to investigate the occurrence of hyperbilirubinemia in a 49-year-old Hispanic man infected with HIV, following an overdose of ritonavir in ritonavir-boosted atazanavir therapy.

**Case presentation:**

A 49-year-old Hispanic man with HIV who had received several highly active antiretroviral therapy regimens over a number of years including atazanavir-containing regimens, was diagnosed with hyperbilirubinemia. An inappropriate doubling of ritonavir boosting resulted in a high atazanavir C_trough _and an initial rise in bilirubin plasma levels. Bilirubin levels later decreased, probably as a consequence of enzyme induction, while atazanavir plasma concentrations remained elevated.

**Conclusion:**

This article describes an occurrence of hyperbilirubinemia in a man infected with HIV and supports the importance of therapeutic drug monitoring in investigations of hyperbilirubinemia among patients receiving antiretroviral agents. That the patient tolerated exceptionally high atazanavir levels further strengthens the tolerability profile of this drug.

## Introduction

The occurrence of hyperbilirubinemia has been reported relatively frequently in clinical trials of the antiretroviral agent atazanavir (ATV). However, hyperbilirubinemia is generally reversible on discontinuation of treatment [1]. Hyperbilirubinemia induced by ATV resembles Gilbert's syndrome [2]. Its symptoms include scleral icterus or jaundice. ATV metabolism involves the enzyme uridine diphosphate glucuronosyltransferase (UGT) 1A1, the activity of which is inhibited by ATV at clinically relevant plasma concentrations [1]. This may lead to a rise in unconjugated hyperbilirubinemia. A significant correlation between serum bilirubin concentration and ATV plasma trough concentration (C_trough_) has been reported by Ray and colleagues in a study of patients receiving either ATV or ritonavir-boosted ATV (ATV/r) [3].

In patients receiving antiretroviral therapy, therapeutic drug monitoring (TDM) may be a useful tool for: (i) assessing the interindividual variability in plasma drug concentrations, (ii) investigating the relationship between drug concentrations and either the anti-HIV effect or drug-related toxicities, (iii) documenting the occurrence of possible drug-drug interactions, and (iv) optimizing therapy in individual patients [4]. We used TDM to investigate the occurrence of hyperbilirubinemia in an HIV-infected 49-year-old Hispanic man with a recent history suggestive of cerebral stroke. The patient had received several highly active antiretroviral therapy (HAART) regimens over a number of years, including ATV-containing regimens.

## Case presentation

We present the case of a 49-year-old Hispanic man with HIV. The patient was diagnosed HIV positive in 1984, the infection most likely resulting from homosexual contact. He was also hepatitis A and hepatitis B positive, was hepatitis C negative and had negative syphilis serology. His total and fractionated bilirubin levels were normal and there was no history of Gilbert's disease in his medical record. In 2001, he suffered an episode of bacterial pneumonia and in 2002 he reached his CD4 count nadir, with 24 cells/mm^3 ^and an HIV-RNA plasma viral load (HIV-RNA pVL) of more than 600,000 copies/ml.

In 2003, he was diagnosed and treated for an oral Kaposi's sarcoma. As a flight attendant, he had a frenetic lifestyle and was treated for HIV infection in different countries. As a result, his medication history included several HAART combinations: (1) tenofovir disoproxil + didanosine + efavirenz (TDF + ddI + EFV); (2) tenofovir disoproxil + zidovudine + lopinavir/ritonavir (TDF + AZT + LPV/r); (3) stavudine + didanosine + lopinavir/ritonavir (d4T+ ddI + LPV/r); (4) stavudine + didanosine + efavirenz (d4T+ ddI + EFV); and (5) didanosine 250 mg once daily + tenofovir disoproxil + efavirenz (ddI 250 + TDF + EFV). The reasons for switching from one regimen to another between 2002 and January 2005 included virological failure, diarrhea and the development of lipodystrophy.

When we first evaluated the patient in January 2005, his CD4 level was 208 cells/mm^3 ^and his viral load was approximately 8000 copies/ml. Genotype testing showed a 190Q mutation related to EFV and a 65R mutation related to TDF. In the protease domain, only a 63P polymorphism was evident.

A new treatment regimen was started of ddI (400 mg once daily), d4T (40 mg twice daily), and ritonavir-boosted saquinavir (SQV/r), 1000 mg/100 mg twice daily. In September 2005, his HIV-RNA pVL was undetectable and CD4 cell count had risen to 232 cells/mm^3^. At this time, his total bilirubin concentration was 1.40 mg/dl (unconjugated bilirubin 1 mg/dl; conjugated bilirubin 0.40 mg/dl). A few weeks later, the patient returned to his home country in South America and was examined again in March 2006, during which his viro-immunological control was close to that obtained in Italy, six months previously.

In May 2006, he returned to our center complaining of colicky abdominal pain and diarrhea with up to eight discharges of loose stools per day. Scleral icterus was noted on physical examination. After excluding drug toxicities, viral hepatitis and alcohol abuse, it became evident that he had been switched from SQV/r to ATV/r while in his native country. Laboratory data showed grade 3 hyperbilirubinemia (total bilirubin 5.4 mg/dl; unconjugated bilirubin 5.1 mg/dl; conjugated bilirubin 0.3 mg/dl) while his HIV-RNA pVL and CD4 count remained basically unchanged. His trough ATV plasma concentration was measured by high performance liquid chromatography (HPLC) and found to be 5.2 μg/ml, around ten times the therapeutic level of 0.15-0.85 μg/ml [5]. The accuracy of drug intake and blood sampling were checked and confirmed as correct.

**Figure 1:**
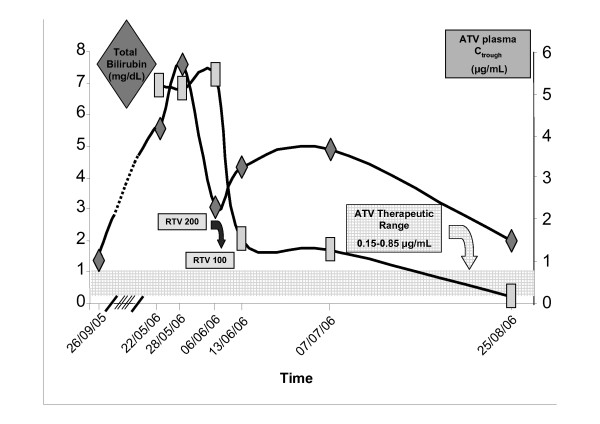
**Total bilirubin and antiretroviral agent atazanavir C_trough _measured from September 2005 to August 2006**.

The patient sought medical care in Milan due to the acute onset of vomiting and diarrhea in May 2006, one week after being examined in our institution. A serum total bilirubin concentration of 7.6 mg/dl (unconjugated bilirubin 7 mg/dl; conjugated bilirubin 0.6 mg/dl) was noted but transaminase levels were within the normal range, as were gamma-GT and alkaline phosphatase. He was discharged from the emergency room with his gastrointestinal symptoms alleviated. In June 2006, the patient returned to our clinic still showing mild scleral icterus but was otherwise asymptomatic. At this stage he presented a note from his medical practitioner from South America that stated he had been taking 300 mg of ATV boosted by 200 mg of ritonavir (RTV). Hence, an incorrect RTV dosage had been taken since May 2006. A further HPLC test in June 2006 confirmed a high level of ATV C_trough _(5.4 μg/ml) (Figure 1). Interestingly, the total bilirubin concentration had spontaneously decreased to around 3 mg/dl. The RTV boosting dosage was reduced to 100 mg, resulting in a decrease of scleral icterus and a total bilirubin concentration of 4.3 mg/ml (unconjugated bilirubin 3.9 mg/dl; conjugated bilirubin 0.4 mg/dl). His ATV C_trough _levels also fell, but were still above the therapeutic range. The patient had subsequent monthly assessments. In July 2006, he presented with mild scleral icterus, a CD4 cell count > 300 cells/mm^3^, and an undetectable viral load. However, his total bilirubin and ATV C_trough _were unchanged.

In August 2006, the patient's jaundice disappeared, his serum total bilirubin concentration was markedly reduced, and his CD4 cell count was unchanged, though levels of HIV-RNA pVL had increased to approximately 200 copies/ml. His ATV C_trough _level was below detection limit (0.1 μg/ml), despite his adherence to the therapy. Thus, both the total bilirubin and the ATV C_trough _decreased after reduction of the RTV boosting dosage. At his last clinic visit in August 2006, the patient's total bilirubin concentration was normal, but the ATV C_trough _was below the therapeutic range. The patient did not return to our clinic and was therefore lost to follow-up.

## Discussion

This case report highlights several points of interest. The inappropriate doubling of RTV boosting resulted in a high ATV trough and an initial rise in the bilirubin plasma level. Bilirubin levels later decreased, probably as a consequence of enzyme induction, while ATV plasma concentrations remained elevated. An "adaptation" in bilirubin levels during ATV-based therapy has been reported indirectly at the 96-week follow-up in the BMS-045 study (120 patients treated with ATV/r), where the incidence of grade 3-4 bilirubin elevation was almost five times higher in the first year of treatment than in the second year (24 versus five cases, respectively) [6]. It is also noteworthy that the patient described here tolerated the high ATV concentrations, and his liver function remained within the normal range throughout the entire period of RTV overdosing. This result provides additional support to the tolerability profile of this drug.

## Conclusion

This article describes an occurrence of hyperbilirubinemia in a man infected with HIV and supports the importance of therapeutic drug monitoring in investigation of hyperbilirubinemia in people receiving antiretroviral agents. That this patient tolerated exceptionally high ATV levels further strengthens the tolerability profile of this drug.

## Abbreviations

ATV: atazanavir; ATV/r: ritonavir-boosted ATV; C_trough_: plasma trough concentrations; HAART: highly active antiretroviral therapy; gamma-GT: gamma-glutamyl transpeptidase; HIV-RNA pVL: HIV-RNA plasma viral load; HPLC: high performance liquid chromatography; TDM: therapeutic drug monitoring; UGT: uridine diphosphate glucuronosyltransferase

## Consent

Written, informed consent was obtained from the patient for publication of this case report and accompanying images. A copy of the written consent is available for review by the Editor-in-Chief of this journal.

## Competing interests

The authors declare that they have no competing interests.

## Authors' contributions

All authors contributed equally to care of the patient. RM wrote the manuscript. All authors reviewed and approved the final manuscript.
